# Victorian paramedics’ encounters and management of women in labour: an epidemiological study

**DOI:** 10.1186/s12884-015-0430-6

**Published:** 2015-02-05

**Authors:** Gayle McLelland, Amee Morgans, Lisa McKenna

**Affiliations:** School of Nursing and Midwifery, Monash University, PO Box 527, Frankston, VIC 3199 Australia; Research Development Manager, Ambulance Victoria, 375 Manningham Road, Doncaster, 3108 Australia; Adjunct Senior Research Fellow, Department of Primary Health Care, Monash University, Frankston, VIC 3199 Australia; School of Nursing and Midwifery, Monash University, Building 13C, Clayton Campus, Clayton, 3199 Australia

**Keywords:** Labour, Birth, Childbirth, Paramedics, Ambulance

## Abstract

**Background:**

Although it is generally accepted that paramedics attend unexpected births, there is a paucity of literature about their management of women in labour. This study aimed to investigate the caseload of women in labour attended by a statewide ambulance service in Australia during one year and the management provided by paramedics.

**Methods:**

Retrospective clinical data collected on-scene by paramedics via in-field electronic patient care records were provided by Ambulance Victoria. Patient case reports were electronically extracted from the Ambulance Victoria’s Clinical Data Warehouse via comprehensive filtering followed by manual sorting. Descriptive statistics were analysed using Statistical Package for Social Sciences (SPSS v.19).

**Results:**

Over a 12-month period, paramedics were called to 1517 labouring women. Two thirds of women were at full-term gestation, and 40% of pre-term pregnancies were less than 32 weeks gestation. Paramedics documented 630 case reports of women in early labour and a further 767 in established labour. There were 204 women thought to be second stage labour, including 134 who progressed to childbirth under paramedic care. When paramedics assisted with births, the on-scene time was significantly greater than those patients transported in labour. Pain relief was provided significantly more often to women in established labour than in early labour. Oxygen was given to significantly more women in preterm labour. While paramedics performed a range of procedures including intravenous cannulation, administration of analgesia and oxygen, most women required minimal intervention. Paramedics needed to manage numerous obstetric and medical complications during their management.

**Conclusions:**

Paramedics provide emergency care and transportation for women in labour. Most of the women were documented to be at term gestation with minimal complications. To enable appropriate decision making about management and transportation, paramedics require a range of clinical assessment skills comprising essential knowledge about antenatal and intrapartum care.

## Background

Although natural, labour is a complex physiological process often lasting many hours before childbirth. Decisions made during labour can directly impact birth outcomes. For many women, clinical onset of early labour can be ambiguous, with women confusing irregular cramps of spurious labour as a sign of established labour, causing apprehension about the best time to seek health care [1].

For a small proportion of women, labour progresses rapidly increasing the possibility of precipitous or unexpected births in the community with higher associated risks [2].

Conversely, premature hospital admission for childbirth has been linked to increased risks of medical intervention due to predetermined progress milestones directed by hospital protocols [3,4]. As a result, labouring women are encouraged to telephone maternity wards prior to hospital attendance to remain at home until labour is established and avoid this ‘cascade of interventions’ [5]. Although midwives find telephone assessment in early labour beneficial, women have expressed dissatisfied with telephone triaging [5,6]. This leaves women wishing to go to hospital with the option of staying at home, making their own way into hospital or calling emergency services for assessment and transport.

Paramedics attend, assist and transport women who have unexpected out of hospital births [2], however, research investigating the women in labour managed by paramedics is scarce. In one ambulance service in the east of England, Foster and Maillardet [7] noted that only one fifth of women transported for imminent birth actually birthed before arrival to hospital, the remaining women were therefore in varying phases of first and second stage of labour. Identifying the changes from the irregular contractions of early labour to commencement of second stage requires specialised clinical skills [8,9]. The challenge of adequate assessment of progress is exacerbated for women who access services not specialising in maternity care. Similar to in-hospital care of women in labour, pre-hospital diagnosis and assessment of progress relies on highly skilled clinical judgement recognising specific cues. Although they are skilled emergency care practitioners, paramedics have limited education underpinning their knowledge of maternity care, with new graduates reporting lack of confidence in managing labouring women [10].

Currently, there is a paucity of research about paramedics’ management of women in labour. This study investigated caseload, clinical features and paramedic care of women in labour for a single calendar year at a state-wide Australian ambulance service.

## Method

### Ethics approval

Ethical approval was obtained from Monash University’s Human Research Ethics Committee and additional approval was provided by Ambulance Victoria’s research committee.

### Study setting including study population

With a land mass of 227,416 square kilometres [11], the Australian state of Victoria has a population of five million people, with three quarters living in the greater metropolitan Melbourne area [12]. Ambulance Victoria provides state-wide emergency healthcare for the Victorian population. In Victoria, the total number of live births increased from 61,108 in 2001 [13] to 72,727 in 2011 [14]. The majority of births (72%) occur in the public health sector with most of the remainder in private hospitals and a small number of home births (0.8%) [14,15]. More than two thirds of all births occur in the greater metropolitan Melbourne area. Very few babies are born in remote rural Victorian maternity services. All complex pregnancies requiring tertiary maternity services are referred to one of three hospitals located in Melbourne [15]. Paramedics are able to consult with a perinatal emergency referral service which provides specialist obstetric advice to all health practitioners dealing with unexpected complex maternity cases [16].

### Data collection

One year of state-wide caseload data was analysed in this study. Retrospective data collected by paramedics between January 1^st^ and December 31^st^ 2009 using an on-scene electronic patient care record information system (VACIS®) was extracted from Ambulance Victoria’s Clinical Data Warehouse. Initially, all males and cases of females aged less than 10 or over 55 years were excluded electronically. Paramedics’ documentation of maternity cases proved to be unsystematic with variables reported in numerous fields or not at all. To confirm that the final dataset included cases related to pregnant and peripartum women, all cases were reviewed manually. The final database included case records for 4096 obstetric related cases and 196 neonates.

**Figure 1:**
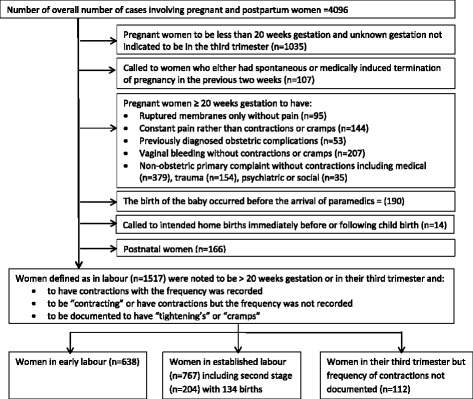
**Selection process of cases from the database involving women in labour.**

Inclusion criteria were cases where paramedics had documented the women at greater than 20 weeks pregnant, or in their third trimester, and experiencing ‘contractions’ or ‘tightenings’ or ‘cramps’, regardless of frequency (Figure 1). Exclusion criteria were cases where paramedics had documented the birth had occurred before their arrival; intended home births immediately before or after childbirth; gestation was less than 20 weeks/second trimester/not documented; pregnancies over 20 weeks gestation without contractions but called paramedics for other reasons including trauma, medical or psychiatric primary symptoms; women who had spontaneous or medically induced termination of pregnancy in the previous two weeks (Figure 1).

### Clinical data definitions

Labour is a continuous process defined physiologically by effacement and dilatation of the cervix [17] which can only be confirmed by vaginal examination, a skill that is outside the scope of practice of Victorian paramedics [18]. For purposes of this study, women noted by attending paramedics as having contractions of any frequency were classified as being in ‘labour’. Active or established labour is clinically recognised by onset of painful regular contractions occurring at least 3 times in 10 minutes [19]. Conversely, women with contractions recorded less frequently than four minutes apart including irregular ‘tightenings’ or ‘cramps’ were considered in early labour. Onset of second stage labour is often signalled by clinical cues including ‘an urge to push’ , ‘anal dilation’ or ‘presenting part on view’ indicating imminent birth [17].

Women with gestations between 20 and 37 weeks were defined as preterm [17,19]. Women with gestations equal to or greater than 37 weeks were considered to have been at term pregnancies.

### Data analysis

Using the clinical signs documented by paramedics, cases were grouped as early and established labour (Figure 1). Descriptive statistical analysis was performed using Statistical Package for Social Sciences (SPSS, v.19). Most continuous variables were not normally distributed, and were analysed using both means with standard deviations and medians with interquartile ranges. Mann–Whitney U tests were performed to determine whether the on-scene time of paramedics was affected by gestation of the pregnancy or stage of labour. Using Chi-square, two comparative analyses were performed. The first compared analgesia administration for women in early and late labour. The second compared paramedic oxygen administration for women at term to preterm labour. Wilcox signed rank tests were performed to investigate changes in initial and final recordings of blood pressure and patient reported pain. For all statistical analyses performed, statistical significance was achieved at p < 0.05.

## Results

Cases were categorised as obstetric/gynaecology for 1421 (94%) patient records (Table 1). Paramedic documentation of clinical features was inconsistent, with data elements recorded in varying fields, including the ‘free text’ field, and some data elements were not recorded.

**Table 1:** **The number of women in labour encountered by paramedics**

	**Number**	**Incidence (%)**	**Definition**
Irregular cramps/tightenings	230	15.2	Early Labour
Frequency of contractions not documented	112	7.4	Early Labour
Contractions > 4 minutes apart	408	26.9	Early Labour
Contractions ≤ 4 minutes apart	563	37.1	Established Labour
Urge to push/anal pouting/perineal dilation	70	4.6	Established Labour/Second Stage
Signs of second stage on arrival resulting in birth	134	8.8	Established Labour/Second Stage
**Total**	**1517**		**100**

The frequency of contractions was recorded in 1405 patient records, with 767 (55%) and 638 (45%) classified as being in established labour early labour respectively. In the established labour group, 204 (27%) women were reportedly in second stage with 134 (66%) assisted by paramedics during childbirth before arriving at hospital.

Other than assessing foetal movements or colour of amniotic fluid, paramedics are unable to monitor the wellbeing of the foetus. As paramedics did not document either of these clinical data elements, no reporting of foetal wellbeing on delivery to hospital is able to be made within this study.

### Demographics

Demographic data are presented in Table 2. Maternal age was recorded in all but two cases. Specific obstetric data including numbers of previous pregnancies (gravida), previous viable births (parity) and gestations were incomplete in many cases (Table 2). Maternal age ranged from 14 to 45 years, with 60% aged younger than 30 years old. One in five women (n = 314, 21%) were in their first pregnancy. Day of the week or month of year did not influence ambulance caseload for this population. However, women were 50% more likely to call paramedics during the night.

**Table 2:** **Maternal Demographic data including scene and transport time for women in labour attended by paramedics**

	**N**	**Min**	**Max**	**Mean**	**Median**	**Q**_**1**_	**Q**_**3**_	**IQR**	**SD**
**Age (years)**	1515	14	45	28.37	28	23	33	10	6.52
**Gestation (weeks)**	1376	20	42	36.65	38.4	35.0	40	5	4.75
**Pregnancy number**	1327	1	17	3.08	3.0	2	4	2	2.15
**Previous Birth**	1321	0	10	1.00	1.0	0	3	3	1.67
*Scene time	1491	0:01:00	1:41:00	0:09:29	0:08:00	0:05:00	0:11:00	0:6:00	0:08:00
*Transport Time	1490	0:01:00	2:58:00	0:19:40	0:17:00	0:11:00	0:25:00	0:14:00	0:13:00

*Scene and transport time are written as hour:minute:second.

*Scene time is the time from paramedics’ arrival at the scene until their departure.

*Transportation time is the time between departure from the scene until arrival at destination.

Gestation of the pregnancy was recorded in most cases (n = 1376, 91%), ranging from 20–42 weeks, with the majority (n = 950, 69%) considered to be at term. Of the women reportedly in preterm labour, 170 (11%) had a gestation between 24–32 weeks. Paramedics noted 35 (2%) women whose pregnancies were less than 24 weeks.

More than three quarters (n = 1177, 78%) of the women lived in the greater metropolitan Melbourne area with another 255 (17%) and 73 (5%) in inner and outer regional centres respectively. There were no women from remote areas of Victoria. When the suburb of residence was compared to the Socio-Economic Indexes for Areas (SEIFA) [20], almost two thirds were classified as socioeconomically disadvantaged areas. Paramedics identified 70 (5%) non-English speaking women, but there may have been more as collection of this information is not compulsory.

### Dispatch and transportation

On receipt of emergency ambulance requests, ambulances are dispatched according to predetermined triage criteria. On 1192 (79%) occasions ambulances were dispatched to cases under the criteria of Labour/Childbirth/Ruptured Membranes. Health practitioners requested the ambulance service to transport women in labour on 69 (5%) occasions. As well as local medical officers requesting paramedics for 34 women from community based clinics, there were eight midwives requiring ambulance transportation to hospital during intended home births. Additionally, 42 (3%) women required inter-hospital transfers including 32 (2%) transported by flight paramedics. The remaining 264 cases (17%) were dispatched as other causes including vaginal bleeding, traumatic injuries, traffic incidents and medical conditions. The majority (n = 1295, 85%) of paramedics were dispatched as high speed or ‘Code 1’ with 187 (12%) as ‘Code 2’ within 25 minutes and 35 (2%) ‘Code 3’ within 60 minutes. Almost all women who called paramedics were transported to hospital, with 26 women (2%) remaining at home.

The on-scene time is from arrival of the paramedics to the time of departure, and was less than 20 minutes for over 90% of cases (Table 2). However, paramedics remained on-scene for one hour or more on ten occasions. Longer on-scene time was required to assist women during childbirth but occasionally paramedics needed to remain for pain management. Transportation time is the length of time between paramedics’ and the patient’s departure from the scene until arrival at the desired destination usually the hospital. Eight out of ten women were transported to their destination in less than half an hour. However, paramedics occasionally needed to spend more than two hours travelling with women in labour (Table 2).

Mann–Whitney U tests were used to investigate scene time for women in early and late labour; term and preterm labour; second stage resulting in birth or second stage without birth under paramedic care. There was no significant difference in scene time for preterm, with median on scene times for both groups 8 minutes (p = 0.53). There was no significant difference in scene time for early and established labour, with median on scene time 7–8 minutes for both groups (p = 0.55). Women in second stage who progressed to childbirth had a significantly longer on scene time (Md = 0:15:30, n = 134) compared to those women who did not progress to childbirth (Md 0:09:00, n = 70), (p < 0.001, r = 0.4).

### Medical complications

Most cases involving women in labour encountered by paramedics were clinically uncomplicated. For some cases, paramedics needed to consider the implications of numerous obstetric, medical and other complications during their management. The most common obstetric complication managed by paramedics was vaginal bleeding, reported in 119 cases (8%) in third trimester and 20 cases (1%) in second trimester. Other obstetric complications included gestational diabetes and pre-eclampsia with one woman having an eclamptic seizure (Table 3). Prior to paramedics’ arrival, a small number of women experienced trauma as detailed in Table 3. Pre-existing medical conditions which potentially impact management were reported including Asthma, Epilepsy and Diabetes Mellitus (Table 3). Whilst paramedics identified only seven women diagnosed with pre-existing hypertension, 250 (17%) women were noted to have initial systolic blood pressures greater than 140 mmHg including 36 (2%) more than 160 mmHg.

**Table 3:** **Complications of women in labour encountered by paramedics**

**Complications**	**Number**	**Incidence (%)**
**Obstetric**		
Gestational diabetes	**38**	**2.5**
Eclampsia	**1**	**0.07**
Pre-eclampsia	**27**	**1.8**
Vaginal bleeding	**144**	**9.5**
**Other medical and trauma**		
Asthma	**127**	**8.0**
Epilepsy	**16**	**0.9**
Diabetes Type 1	**6**	**0.5**
Diabetes type 2	**7**	**0.6**
Hypertension	**7**	**0.5**
Trauma	**14**	**0.9**
**Mental health and substance misuse**		
Anxiety	**13**	**0.9**
Bipolar disorder	**7**	**0.5**
Depression	**46**	**3.0**
Schizophrenia	**2**	**0.13**
Alcohol	**2**	**0.13**
Cannabis	**11**	**0.7**
Heroin	**14**	**0.9**
Methodone	**19**	**1.3**

Paramedics reported 59 (4%) cases involving women with mental health issues including eight who had been previously diagnosed with multiple conditions (Table 3). Paramedics also reported encountering 48 (3.0%) women with previous histories of substance misuse, including 13 currently taking combinations of various drugs (Table 3).

### Paramedic management

The majority of women (n = 926, 61%) did not require any interventions. Paramedics performed a variety of procedures for the remaining cases detailed in Table 4. Methoxyflurane was the primary analgesia of choice, with administration to 573 (38%) of women. Five women required more than one type of pain relief.

**Table 4:** **Procedures performed by paramedics**

**Procedures and medications**	**Number**	**Incidence (%)**
**Procedures**		
Advice given	**14**	**0.9**
Auto infusion	**13**	**0.9**
Cardiac monitor	**164**	**10.8**
Haemorrhage control dressing	**14**	**0.7**
IV Normal Saline Flush	**24**	**1.6**
IV Therapy	**30**	**2.0**
No interventions performed – just transport	**900**	**59.3**
Oxygen therapy	**173**	**11.4**
Pulse oximeter	**28**	**1.8**
Rest and reassurance	**1321**	**87.1**
**Medications given**		
IV Fentanyl	**1**	**0.07**
Morphine	**7**	**0.5**
Methoxyflurane administration	**582**	**38.4**
Metoclopramide	**2**	**0.07**
Midazolam	**1**	**0.07**
IV Prochlorperazine	**1**	**0.1**
Salbutamol	**3**	**0.2**

Chi-square tests for independence were conducted to explore oxygen administration in term and preterm labour; analgesia administration in early and established labour; analgesia administration in established labour and second stage. A total of 173 women received oxygen, with paramedics were significantly more likely to administer oxygen to women in preterm labour (p < 0.001). Less than 40% of women received pain relief, however paramedics were significantly more likely to administer analgesia to women in established labour (p < 0.001). A Chi-square analysis indicated no significant association between pain relief in established labour and second stage (p = 0.33).

Wilcox Signed Rank Tests were conducted to evaluate change between initial and final blood pressure readings and pain scores. There was a statistically significant decrease in the final blood pressure readings (p < 0.001) with small to medium effect (r = 0.2). There was a statistically significant decrease in pain scores (p < 0.001) with the median final pain score recorded as 5/10.

## Discussion

This study explored paramedics’ management and transportation of women in labour. Albeit a relatively small proportion of ambulance service workload [21], results clearly demonstrate that Victorian paramedics encountered approximately 30 women in labour per week. The majority of these women were considered to be at term gestation and their management was clinically uncomplicated. One in five women attended by paramedics were experiencing their first pregnancy. Compared to the overall maternal age in the year of this study [22], the women in labour encountered by paramedics were generally younger. A small proportion of women had complications potentially impacting upon their labour which paramedics needed to consider during management.

Victorian paramedics work within a Clinical Practice Guideline (CPG) framework to make clinical decisions about management and conveyance to hospital in appropriate timeframes for each patient [21]. Overall Victorian paramedics transport 90% of clients to hospital [21]. Nearly all women (98%) in labour were transported within time-frames recommended by Ambulance Victoria.

Women in labour, and those supporting them, can present as vulnerable and anxious, often seeking reassurance from midwives and other health professionals [1,4], potentially motivating them to call emergency services. Once in attendance, confirmation of physiological progress of labour is not possible for paramedics but their decisions and management can substantially impact the woman’s experience when considered within the ‘cascade of interventions’ framework, which indicates that women at term would be at increased risk of interventions including epidural and caesarean section [3,4] when transported to hospital in early labour. However, women in preterm labour may require assessment or monitoring regardless of the stage of labour. Without appropriate skills and knowledge to perform an adequate assessment of the presenting clinical symptoms, paramedics have little choice but to transport women in labour regardless of labour progression or gestation.

Paramedics were required to assist women in imminent birth either transitioning from first to second stage, or actively in second stage, with nearly two-thirds in second stage progressing to childbirth under paramedic care. Most unexpected births managed by paramedics are precipitous and uneventful, but the outcomes are generally poorer for both mother and baby compared to either in hospital and planned home births [2]. Whilst assessing progress in labour may be difficult for paramedics, they responded to the women’s needs for pain relief with significantly more women in established labour receiving analgesia. However, there was no statistical difference in analgesia received by women in second stage compared to those in established labour which may affect the outcomes for mother and baby [17]. Without knowledge of clinical cues, assessing progress into second stage of labour is difficult for paramedics, impacting clinical decision making between transportation or remaining at the scene in anticipation of childbirth. With the on-scene time for the women who progressed to birth significantly greater than for those who were transported to hospital, the results suggest that paramedics recognised the signs of imminent birth and chose to stay on scene rather than risk childbirth en route to hospital.

As well as understanding women in uncomplicated labour, paramedics require substantial knowledge regarding the implications of preterm labour, which was recorded in a third of cases, almost half at gestations likely to require neonatal intensive care resources [15]. With all tertiary centres located in metropolitan Melbourne, transporting from outer metropolitan, regional and rural areas requires astute clinical judgement when deciding the safest transport options.

Paramedics are skilled emergency care practitioners who provide care to a wide range of the population for multiple ailments, with maternity care a small proportion of their workload. Victorian paramedics are able to consult with referral services for perinatal emergencies [16] encountered, however there is no evidence in this study that these resources were used. Whilst consulting a perinatal emergency service may be beneficial, paramedics require specific maternity clinical knowledge to recognise the indications to accurately assess and report to the service, and then implement advice given. New graduate paramedics recruited by Ambulance Victoria undergo emergency maternity care instruction within undergraduate programs. However, this content varies between universities and is often difficult to ascertain because only a few offer standalone maternity education content [23,24], with the majority including the content in life span or special population units [25-28].

### Limitations

This research was conducted on data entered into an information system by Victorian paramedics at time of treatment. The data are collected for treatment rather than research purposes and therefore some key elements of missing. Similar issues with incomplete documentation by paramedics have been reported internationally [29,30] when investigating paramedics’ encounters with unexpected out of hospital births. Another limitation of this research is the inability to evaluate the outcomes of the women following admission to hospital. Further research into paramedics’ management of women in labour is recommended, particularly the establishment of a minimum dataset.

## Conclusion

Paramedics are required to use substantial but specific childbirth clinical management skills and knowledge to make treatment and transport decisions for women in labour. Women in labour are a vulnerable population relying upon decisions made by health professionals including paramedics. Paramedics require adequate education in obstetric care and their repertoire of skills must include the ability to care for women in various stages of labour and understand and communicate effectively with the available maternity support and referral services.
